# Schizophrenia, bipolar disorder, or intracranial aneurysm? A case report

**DOI:** 10.1002/brb3.2245

**Published:** 2021-07-21

**Authors:** Yifan Shi, Yezhou Tang, Zhiguo Wu, Jun Chen, Jia Huang, Yiru Fang

**Affiliations:** ^1^ Division of Mood Disorder Clinical Research Center Shanghai Mental Health Center Shanghai Jiao Tong University School of Medicine Shanghai China; ^2^ Department of Psychiatry and Neuropsychology Shanghai Deji Hospital Qingdao University Shanghai China; ^3^ CAS Center for Excellence in Brain Science and Intelligence Technology Shanghai China; ^4^ Shanghai Key Laboratory of Psychotic Disorders Shanghai China

**Keywords:** bipolar disorder, intracranial aneurysm, schizophrenia, subarachnoid hemorrhage

## Abstract

**Background:**

Mental disorders are a common finding among patients with unruptured intracranial aneurysms.

**Case:**

The current case concerns a young man with an anterior communicating artery aneurysm who was misdiagnosed with schizophrenia and bipolar disorder due to his significant psychosis and mood episodes. Having undergone surgery on the unruptured intracranial aneurysm, the patient's psychiatric symptoms disappeared, and he maintained a stable mood during the 3‐year postoperative period.

**Discussion:**

The case is indicative of the need to consider the possibility of organic brain lesions in patients with first episodes of psychiatric presentations.

## Graphical abstract

We describe a young man with anterior communicating artery aneurysm who was misdiagnosed as schizophrenia and bipolar disorder due to his significant psychosis as well as mood episodes and got totally recovered from his psychiatric conditions and maintained a stable mood after an operation on unruptured intracranial aneurysm in the following three‐year. The case suggests that organic brain lesions in patients with first episode of psychiatric presentations should be carefully considered.

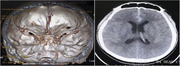

## 1 CASE PRESENTATION

Mr. Z is a right‐handed, 28‐year‐old young male. His symptoms were first manifested at the age of 17, when he complained of headache that felt like lava spreading out within his head. He reported this to his parents. In addition, he also began to experience paranoia, manifest in the proposition that one of his teachers was monitoring him. This manifestation was assumed to be related to the combined pressures of school life and homework. Specifically, the patient regarded the telephone as a monitoring device controlled by a particular teacher. In addition, his constant suspicion that he was under criminal investigation placed him in a perpetual state of fear. Overall, his sense of persecution caused him to feel stressed and unhappy at school. These feelings were expressed as hostility toward his teacher and a refusal to attend school. His parents felt that his behavior was abnormal and took him to a psychiatric clinic where he received a diagnosis of schizophrenia (based on ICD‐10 criteria) and was subsequently treated with mirtazapine (up to 90 mg/day) and quetiapine (up to 300 mg/day).

However, after 1 year of treatment with medication, neither the paranoia nor the headaches had improved. Moreover, the patient had begun to believe that he was also being monitored by his neighbors and strangers. In addition, he now presented with an elated mood, increased energy and activity, flight of fancy, and elevated distractibility almost every day for a period of 1 month, which resulted in failed relationships with his schoolmates. Two months later, he experienced an episode wherein he demonstrated a period of uninterrupted anxiety and irritability, accompanied by feelings of worthlessness, loss of pleasure, and a diminished ability to think. He also complained repeatedly about the swollen pains in his head. The diagnosis was revised as bipolar disorder (based on ICD‐10 criteria) by another psychiatrist, after which a combination therapy of sodium valproate (up to 1000 mg/day) and olanzapine (up to 10 mg/day) was commenced. The mirtazapine therapy was discontinued, while the dosage of quetiapine was reduced to 100 mg/day. His psychiatric symptoms disappeared and his emotional stability and headache severity gradually improved after 6 months with the new therapeutic regimen. However, the patient continued to experience difficulties with his thoughts and memories. Moreover, he developed an unusual strong interest in pornography and easily became extremely excited when talking with his female classmates. Believing himself to be cured, the patient discontinued his medication when he was 19, without consulting his psychiatrist. Due to his poor performance in the National Higher Education Entrance Examination, he was obliged to attend a junior college instead of a university. Subsequently, he struggled to complete his studies at junior college and became an office worker. He continued to suffer from repeated, albeit less severe mood swings and paranoia following a renewed onset of minor headache. Moreover, he did not have any period of emotional stability for more than 3 months. Consequently, he found it challenging to complete tasks and maintain relatively harmonious relationships with others.

Eight years after the initial onset of his delusions and 7 years after the first episode of detectable mood disorder, he attended an emergency department at age 25, having experienced a sudden onset of severe headache and nausea.

**FIGURE 1:**
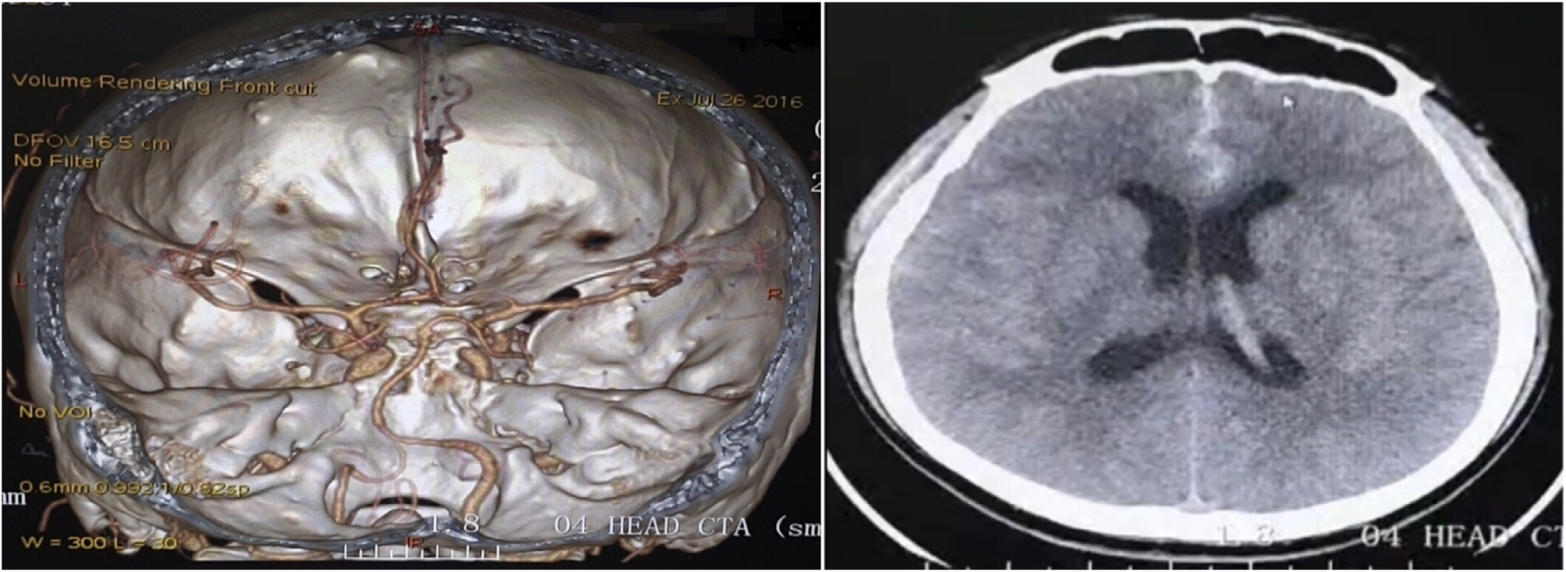
Computed tomography angiography revealed an anterior communicating aneurysm with a subarachnoid hemorrhage

The physical examination performed upon admission found the patient to possess a painful demeanor. He was clearly conscious, with stable vital signs, and regular and coordinated limb movement. Both pupils were equal and reactive. His deep tendon reflexes were 2+ and equal on both sides without pathological reflexes. Cerebrospinal fluid examination revealed a red blood cell count of 3200 × 10^6^ cells/L and a white blood cell count of 240 × 10^6^ cells/L. Pandy's test indicated a positive result, while liver function, renal function, and serum electrolyte test were all normal. Cerebrospinal fluid culture was sterile after 5 days of incubation. However, computed tomography angiography of the head revealed a 2.1 mm × 4.8 mm aneurysm in the anterior communicating artery with a subarachnoid hemorrhage (Figure 1).

The clinical diagnosis of subarachnoid hemorrhage as a consequence of an anterior communicating aneurysm was made based on the patient's clinical presentation, laboratory tests, and the imaging examination. Following consultation with several neurosurgeons who were on duty that day, the patient underwent emergency digital subtraction angiography and Guglielmi detachable coil embolization, which were successful.

After the operation, the patient's symptoms, previously ascribed to schizophrenia and bipolar disorder, did not reemerge during the course of a 3‐year follow‐up without the use of mood stabilizers and antipsychotics. His mood stability, energy levels, cognitive status, and other symptoms returned to normal, and he no longer complained about his headache. He exhibited good interpersonal skills and a capability for employment in an office job.

## 2 DISCUSSION

Intracranial aneurysms are estimated to have a prevalence of 3.2% of the adult population worldwide, with peak onset between 40 and 60 years of age. Intracranial aneurysms are acquired lesions that are caused by degenerative changes in vessel walls. Some increase in size over a period of hours, days, weeks, or several years, and eventually either rupture or undergo stabilization and hardening. Most intracranial aneurysms are asymptomatic prior to rupture, although some patients may present with symptoms such as headache, transient ischemic attack, cranial neuropathies, double or blurred vision, and seizures (Thompson et al., 2015).

The early detection of unruptured intracranial aneurysms continues to present a challenge due to their nonspecific physical and psychological symptoms. As such, patients with unruptured intracranial aneurysms are occasionally misdiagnosed with psychogenic disorders.

Prior to the aneurysm rupturing, the patient in the current case presented with sporadic, endurable headaches, no visual irregularities, and no pyramidal dysfunction. However, the patient had experienced discernable mood swings and feelings of paranoia. The combined administration of mood stabilizers and atypical antipsychotic drugs caused both the headaches and psychological symptoms to improve. The psychiatrists’ focus on the psychiatric symptoms exhibited by patient Z caused the progress of the physical manifestations to be overlooked. Consequently, neither the psychiatrist nor the patient requested any imaging examination. Had they done so, the root cause of the symptoms would become clear. However, the patient's underlying illness remained undiagnosed.

A review of the treatment process suggests that several stages in the treatment process should be reviewed. First, the patient initially presented with headaches for which there was no clear cause. Rather than assuming that the headaches were a symptom of a psychiatric illness, it would have been preferable to have conducted cranial imaging evaluation at this early stage in order to rule out any organic abnormalities. Second, throughout the course of the disease, the patient's mental symptoms and headaches always occurred concurrently. The psychiatrist treated the patient with valproate, which is reported to be effective in preventing migraines, with the dosage being in the range of 500–1500 mg/day (Pringsheim et al., 2010). Therefore, even if the patient's headache reduced after using the drug, it was not possible to conclude that the improvement of the bipolar disorder symptoms was the cause of the headache relief. Third, the patient presented an inadequate response to effective doses of mood stabilizers and second‐generation antipsychotics. He also showed confabulation, changes in personality, and impairment in memory, learning, attention, and executive functions, all of which are consistent with the neurobehavioral disturbances associated with intracranial aneurysms (Bottger et al., 1998). Consequently, the diagnoses of schizophrenia and bipolar disorder should have been questioned.

How the intracranial aneurysm caused mania, paranoia, and other psychiatric symptoms in this patient remains unclear. It is possible that the intracranial mass effect and ischemic changes caused by the intracranial aneurysm, in addition to the inflammatory response related to the formation, growth, and rupture of the intracranial aneurysm might have possibly led to Mr. Z's mood instability and other psychiatric presentations.

This study has certain limitations. For example, the patient's intracranial aneurysm, mental symptoms, and psychiatric symptoms may have recovered spontaneously. However, it is difficult to draw any absolute conclusion due to the lack of detailed imaging data. Although no such case has previously been reported, this paper suggests that this possibility should be considered.

Misdiagnosis of functional mental disorders may result in the failure to recognize and treat neurosurgical disorders in a timely manner. Several critical issues must be addressed in order to avert similar misdiagnoses, the first of which is that the somatic and psychological symptoms of patients must be accorded equivalent credence. Second, possibility of organic encephalopathy for first‐onset cases must be heeded. Third, careful assessment must be made of both the course of illnesses and patients’ responses to routine treatment. Furthermore, Madhusoodanan et al. have recommended neuroimaging for patients who present with one of several clinical conditions, to wit: new‐onset psychosis, new‐onset mood/memory symptoms, the occurrence of new or atypical psychiatric symptoms, and anorexia without body dysmorphic symptoms (Madhusoodanan et al., 2007). Adopting these measures may help to ensure that the possibility of intracranial organic lesions can be excluded as a cause of symptoms prior to the diagnosis of a mental disorder.

The specific clinical manifestations of patient Z also emphasize the importance of individualized diagnosis. Current research indicates that normative models can effectively reveal heterogeneous biology at an individual level (Marquand et al., 2019). Healthy individuals usually do not deviate significantly from the normative model. Patients with schizophrenia typically demonstrate significant reductions in gray matter in their frontal lobes, cerebellum, and temporal cortex. Conversely, patients with bipolar disorder present with differences that are primarily confined to cerebellar regions (Wolfers et al., 2018).

In summary, the psychiatric symptoms caused by anterior communicating artery aneurysms could present in a manner akin to those caused by either schizophrenia or bipolar disorder. This case highlights the need for an elaborate differential diagnostic process, such as brain imaging, in cases where headaches are among the initial symptoms.

## CONFLICT OF INTEREST

The authors declare that there is no conflict of interest.

### PEER REVIEW

The peer review history for this article is available at https://publons.com/publon/10.1002/brb3.2245.

## Data Availability

The data that support the findings of this study are available from the corresponding author upon reasonable request.
